# Gene processing control loops suggested by sequencing, splicing, and RNA folding

**DOI:** 10.1186/1471-2105-11-602

**Published:** 2010-12-20

**Authors:** Clark D Jeffries, Diana O Perkins, Xiaojun Guan

**Affiliations:** 1 Eshelman School of Pharmacy and Renaissance Computing Institute, University of North Carolina at Chapel Hill, NC, USA; 2 Renaissance Computing Institute, University of North Carolina at Chapel Hill, NC, USA; 3 Department of Psychiatry, University of North Carolina at Chapel Hill, NC, USA; 4 Center for Bioinformatics, University of North Carolina at Chapel Hill, NC, USA

## Abstract

**Background:**

Small RNAs are known to regulate diverse gene expression processes including translation, transcription, and splicing. Among small RNAs, the microRNAs (miRNAs) of 17 to 27 nucleotides (nts) undergo biogeneses including primary transcription, RNA excision and folding, nuclear export, cytoplasmic processing, and then bioactivity as regulatory agents. We propose that analogous hairpins from RNA molecules that function as part of the spliceosome might also be the source of small, regulatory RNAs (somewhat smaller than miRNAs).

**Results:**

Deep sequencing technology has enabled discovery of a novel 16-nt RNA sequence in total RNA from human brain that we propose is derived from RNU1, an RNA component of spliceosome assembly. Bioinformatic alignments compel inquiring whether the novel 16-nt sequence or its precursor have a regulatory function as well as determining aspects of how processing intersects with the miRNA biogenesis pathway. Specifically, our preliminary in silico investigations reveal the sequence could regulate splicing factor Arg/Ser rich 1 (SFRS1), a gene coding an essential protein component of the spliceosome. All 16-base source sequences in the UCSC Human Genome Browser are within the 14 instances of RNU1 genes listed in wgEncodeGencodeAutoV3. Furthermore, 10 of the 14 instances of the sequence are also within a common 28-nt hairpin-forming subsequence of RNU1.

**Conclusions:**

An abundant 16-nt RNA sequence is sourced from a spliceosomal RNA, lies in a stem of a predicted RNA hairpin, and includes reverse complements of subsequences of the 3'UTR of a gene coding for a spliceosome protein. Thus RNU1 could function both as a component of spliceosome assembly and as inhibitor of production of the essential, spliceosome protein coded by SFRS1. Beyond this example, a general procedure is needed for systematic discovery of multiple alignments of sequencing, splicing, and RNA folding data.

## Background

The numerous, very dissimilar types of bioinformatic data conspire to make integration a central problem for efficient and effective application of biological findings. Integration of data of three particular types is the goal of this paper. Gene splicing is the focus, held up as an example of how sequencing, splicing, and RNA folding data types might be used to guide research that could illuminate major mechanisms of cell biology such control of levels of ribonucleoprotein species.

Function and dysfunction of gene splicing impact embryogenesis, cell motility and viability, cell cycle arrest, and many other mechanisms of metazoan cell biology [1]. This paper stems from three remarkable observations involving splicing. The spliceosome is a large complex of protein subunits and five ribonucleoprotein subunits, the latter incorporating snRNAs. One of the snRNAs is the 164-nt RNU1. Predicted 2D molecular shapes of RNU1 include four "hairpins," conformations in which pairs of nucleic acids form a double-stranded stem while single-stranded nucleic acids form a loop. The first two of the RNU1 hairpins are already known to be bioactive through functional assays of regulation of the gene cyclin H (CCNH) [2]. The fourth hairpin, denoted herein as **H**, has a loop of four nts and a stem of 12 pairs of nts including eight C-G bonds (hence is very stable).

Our deep sequencing to detect small RNAs in three samples of post-mortem human prefrontal cortex produced abundant reads corresponding to a 16-nt sequence from the 3' side of the stem of **H**. We denote herein the 16-nt sequence as **S**.

Regarding small RNA context [3], Kawaji et al. engaged in unbiased exploration of 19- to 40-base sequences from small RNAs. Their pioneering report provided evidence of abundant small RNAs originating from familiar noncoding RNAs (ncRNAs) including tRNAs, snoRNAs, snRNAs, and rRNAs. Regarding tRNAs, 3' ends fragments are transported from the nucleus to accumulate in the cytoplasm, as reported by Liao et al. [4]. Bidirectional promoters suggested that small RNAs can be derived from double stranded RNAs (dsRNAs) with subsequent cleavage. Shi et al. [5] found abundant transcriptional representation of sequences immediately adjacent to--that is, offset from--predicted pre-miRNAs in the simple tunicate *Ciona intestinalis *(sea squirt). Langenberger et al. [6] also found transcripts offset from miRNAs in human samples, albeit at low levels unrelated to levels of the adjacent miRNAs. Taft et al. [7] first reported ~18 nt RNAs in FANTOM4 data that map within -60 to +120 nt of transcription start sites of genes of humans and other metazoans. Taft et al. [8] then found miRNA-like small RNAs derived from the ends of snoRNAs in humans and other eukaryotes. Moreover, Taft et al. [9] reported 17- or 18-nt RNAs with 3' ends that map precisely to the splice donor site of internal exons of mice and other metazoans. Regarding snoRNAs, Ender et al. [10] assayed human cancer cell RNAs and reported a number of human snoRNAs with miRNA-like processing signatures, evidently targeting an mRNA. Likewise, Saraiya et al. [11] used sequencing to find a 26-nt RNA from the flagellated protozoan *Giardia lambia*, again with miRNA-like processing and apparent RNAi activity. Other non-miRNAs of about 16 nts that are subsequences of known miRNAs have been shown by Li et al. to participate in gene regulation, targeting the 3'UTRs of target genes as efficiently as sequentially enclosing miRNAs [12]. Importantly, Li et al. documented a long list of small RNAs, some with known sources and some not. In a generalising study, Langenberger et al. [13] discovered from sequencing data that certain small RNA subsequences of a variety of human ncRNAs are highly overrepresented in the transcriptome, extending all the above reports. They analysed low molecular weight RNAs isolated from frozen prefrontal cortex, as did we in preparation of the present report. A rapidly developing line of research on small RNAs derived from tRNAs is represented by work of Haussecker et al. [14].

Additional sources of small ncRNA are the vault RNAs, ~100-nt Pol III transcripts in the enigmatic vault organelles of eukaryotic cells. There are three described human vault RNAs from a cluster on chromosome 5 [15]. Stadler et al. [16] reported differential vault RNA expression in five human cancer cell lines and consensus patterns of small RNAs from vault RNAs across species. Vault particles are associated with multidrug resistance and intracellular transport. Persson et al. [17] discovered that human vault RNAs produce several small RNAs via mechanisms different from the canonical miRNA pathway, but at least one such small RNA associates with Argonaute proteins and guides sequence-specific cleavage of mRNAs to regulate gene expression. In particular Persson et al. discovered regulation of CYP3A4 (one of 57 human cytochrome P450 proteins) in MCF7 cells by a small byproduct of vault RNA transcription. The CYP3A4 enzyme is important in the initial metabolism of many marketed drugs [18]. Importantly, the experiments of Persson et al. might explain the association of abundance of vault particles with drug resistance.

It seems quite likely that nature must put such abundant, selected subsequences of the above types to some purpose, implying unrevealed pathways that are presently without definitive annotations or even realisation [3]. For example, nuclear-localized small RNAs might be epigenetic regulators of gene expression [9]. Thus block patterns of small RNA transcription sources might greatly improve and simplify ncRNA annotation [13].

Regarding neurological bioactivity, Smalheiser et al. [19] discovered in adult mouse hippocampus that certain species of 25- to 30-nt small RNAs derived from specific sites within well known noncoding RNAs were dramatically increased as a consequence of odorant discrimination training. This work reveals the potential importance of byproducts of ncRNA synthesis in neuroscience, possibly a universe of gene regulation parallel to that of the miRNAs.

Consistent with the above prior work, we found that reads representing the 16-nt sequence **S **appear in every sample more than ten times as frequently as reads from the other three RNU1 hairpins and at frequencies comparable to those of abundant brain miRNAs. Further compounding interest in the 16-nt sequence **S **from hairpin **H **are, in the manner of miRNA target predictions, two putative target regions (lengths 9 and 11 nts) in the 3'UTR of splicing regulator gene SFRS1. Thus the 16-nt byproduct of RNU1 synthesis (from promotion of splicing) might also inhibit expression of SFRS1 (inhibition of splicing or at least inhibition of formation of spliceosome components). This might be a form of auto-regulation essential to homeostasis of splicing. Our neuroscience interests provide focus on SFRS1 protein product because it modulates several forms of synaptic plasticity considered to be involved in the very essence of memory [20].

Thus there is a triple intersection of bioinformatics: annotated function of an ncRNA, abundance in brain of a small RNA evidently processed from the same ncRNA source, and sequence alignment of the complement of the same small RNA with the 3'UTR of a major gene having the same function. These *in silico *coincidences demand investigation of potential miRNA-like mechanisms involving the RNU1 hairpin **H**, especially with regard to SFRS1. Needed are functional validations of nuclear RNU1 targets. Considering the huge impact of splicing function in nature and dysfunction in disease, elucidation of splicing homeostasis would carry a significant potential for progress toward novel diagnostic tools and drug platforms.

Regarding RNU1 context, hairpins studied by O'Gormann et al. [2] (which do not include **S**) were found to be bioactive, as mentioned above. Additionally, it has long been known that pre-mRNA splicing can be regulated both positively and negatively by reversible phosphorylation of spliceosomal SR proteins [21,22]. Thus it would be no surprise that additional layers of complexity might exist to regulate bioactivity of SFRS1 protein. Moreover, Kohtz et al. [23] showed at an early date that SFRS1 protein cooperates with U1 small nuclear ribonucleoprotein particle (snRNP) in binding pre-mRNA, so there is already a direct, mechanistic link of RNU1 in U1 with SFRS1 protein. However, demonstrating that a small RNA byproduct of RNU1 transcription goes on to bind to SFRS1 mRNA and inhibit expression of that gene would be, to our knowledge, a novel splicing feedback loop discovered by virtue of modern, unbiased sequencing.

In summary, alignments of abundant reads, hairpin structures, and logical targets are known to be important in some cases and as yet unrecognised alignments are likely to be important in others--provided such colligations can be efficiently discovered.

## Results and discussion

We note that the topic of processing small RNA sequencing results is a very active area of research with several important, powerful search and alignment engines developed along lines of analysis somewhat different from ours. These are represented by development of software for efficient and fast selection of abundant core sequences within numerous short reads by Hoffmann et al. [24], the description of novel miRNA discovery methods with mirTools from Friedländer et al. [25], and comprehensive statistical and annotative methods in mirTools by Zhu et al. [26] and Gunaratne et al. [27].

The conformations of the full RNU1 molecule are predicted by mfold [28] to include four hairpins, of which the 3' end hairpin **H **is of interest due to sequencing abundance and high predicted hairpin stability. The nascent RNU1 transcript is presumably chaperoned by proteins, but this hairpin might be so stable that it immediately forms and remains folded in most or all RNU1 conformations. Regardless, the strong signal for **S **in RNA from human brain and from our custom assays of human neural stem cells suggests that some mechanism isolates **S **from the hairpin **H **and protects it as a 16-nt single-stranded RNA from digestion.

**Figure 1:**
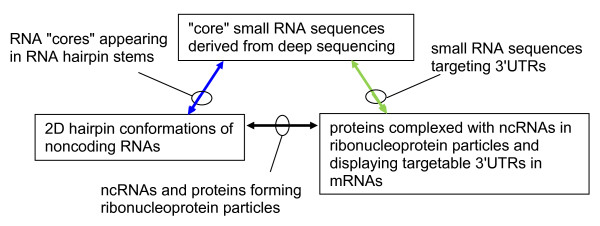
**Three types of bioinformatic data linked pairwise in a triangle**. "Core" RNAs are short sequences appearing abundantly in intersections of sets of deep sequencing reads. Noncoding RNAs (ncRNAs) generally have predicted 2D structures and certain well-formed hairpins of some 20 to 100 nt, possibly with cores embedded in stems. The same ncRNAs can also be associated with proteins in ribonucleoprotein particles (RNPs). Lastly, mRNAs coding the same RNP proteins have 3'UTR regions that might be targeted in an miRNA-like manner by short RNAs, including some core RNAs.

In summary, we advocate development a rigorous methodology leading to the general discovery of multiple alignments of the **S **type as depicted in Figure 1.

## Conclusions

As expressed by Kawaji et al. [3], nature seems to shun dogmatic classification of small biological RNA molecules. They point out that the likelihood of unrevealed pathways, implied by discovery of abundant small subsequences from particular regions of ncRNAs, requires us to avoid premature definitions, specifications, and annotations of the transcriptome and its regulation. The present paper reports a remarkable colligation, a suggestive triple alignment of disparate bioinformatic observations, and it points to a line of investigation regarding the stable abundance of an organelle that is a hallmark of metazoan diversity, the spliceosome. What other colligations that include ncRNA fragments remain to be discovered? How can they be efficiently and systematically discovered in the contexts of other organelles and cell functions? These questions, partly answered by the research of others and the present work, demand attention and resources appropriate to elucidation of the foundations of cell biology.

## Methods

We have sequenced Dicer-processed small RNAs from dorsolateral prefrontal cortex (Brodmann area 9) of three persons who had no mental illness at time of death. Samples were generously made available from the Stanley Medical Research Institute. The spliceosome is involved in synaptic plasticity, critical to normal brain function. It is of interest that altered expression of SFRS1 has been reported in post-mortem schizophrenia studies involving variable isoforms of DISC1 [29] or NCAM1 [30].

We discovered a 16-nt sequence--herein denoted as **S **= TTCGCGCTTTCCCCTG or UUCGCGCUUUCCCCUG--in thousands of reads, and we confirmed by PCR the presence of **S **in commercially available neural stem cells derived from human embryonic stem cells. **S **includes 9 and 11 nucleotide subsequences that appear exactly as the reverse complements of two subsequences of the 3'UTR of the important splicing gene SFRS1. Furthermore, **S **is included as part of the loop and the 3' side of a well-formed 28-nt hairpin **H **from the snRNA RNU1. Parallel to other studies cited above, this level of complementary suggests that **S **could bind to the 3'UTR of SFRS1 and inhibit processing or accelerate sequestration or degradation of SFRS1 mRNA, possibly in an miRNA-like manner.

### Methods of sequence analysis

As yet undiscovered regulatory small RNAs might function in normal human brain or might provide signatures of human brain diseases. Total RNA from dorsolateral prefrontal cortex was isolated using Trizol under the auspices of Stanley Medical Research Institute. We derived cDNA libraries using the Illumina Small RNA Sample Preparation Kit (San Diego CA), following the manufacturer's version 1.5 protocol. The cDNA libraries were sequenced with an Illumina Genome Analyzer at our university core facility. Resulting 35-base reads were first filtered to remove concatenated adaptors at the 3' ends. Specifically, we sought the first six or more bases of the Illumina 3' adapter TCTCGTATGCCGTCTTCTGCTTGAAA... in valid reads. Next a variable number of consecutive As were trimmed from 3' ends. Those remaining sequences with 16-29 bases were aligned with ClustalW2 [31] and then further filtered by requiring at least three exact copies. Frequently appearing subsequences of length ≥16 bases were designated "core sequences," including **S **itself. Our algorithm was a modification of published methods [12,32]. Sources of cores from all three subjects were about 64% from mature miRNAs, 27% from unknown transcripts, 4% from ends of tRNAs, 3% from snoRNAs, 1% from snRNAs (including **S**), and 1% from mitochondrial tRNAs.

We obtained raw read counts from three subjects as follows: 1,813,994; 2,276,814; 3,655,462. Counts of distinct core sequences were 1213, 1423, 1790. Counts of **S **itself were 4736, 1317, 2453.

We generated 1000 distinct, random permutations of the 16 bases corresponding to **S **and sought them among all raw reads of all three samples. Not one was found. The probability of finding not even one of 1000 16-base random sequences within a million, distinct, random, 30-base sequences is 0.0304 (but of course read sequences are not random). Thus while **S **itself is plentiful among our reads, distinct permutations of the bases corresponding to **S **yield random sequences that are consistently absent.

We used BLAT in UCSC Human Genome Browser and found full 16-base sources of **S **to occur only when preceded by TGCA or TGCG and only in 14 genomic locations, all 14 listed within the RNU1 genes in wgEncodeGencodeAutoV3.

Given our interest in neuroscience, we sought to determine if human embryo-derived neural stem cells also generated copies of **S**. Cells used in these experiments were commercially available neural stem cells called hNP1 cells from ArunA Biomedical (Athens GA) derived from human embryonic WA09 cell lines. hNP1 cells grow as an adherent monolayer and are homogeneous, uniformly expressing various neural stem cell proteins (e.g. NES, SOX2) and low levels of embryonic stem cell proteins (e.g. POU5F1 (alias OCT4)) [33,34]. hNP1 neural stem cells are subject to rigorous quality control including DNA fingerprinting, viral testing, and maintenance of a stable karyotype.

We cultured hNP1 cells according to the manufacturer's protocol. We harvested about 1 million passage 4 cells. Cytoplasm and nuclear lysate were separated using the Norgen Cytoplasmic and Nuclear RNA purification kit (Thorold ON). We verified partitioning with an Agilent Bioanalyzer (Santa Clara CA) by observing with a DNA chip a DNA:DNA concentration ratio of >100X of nucleus over cytoplasm and for the same samples processed with a Bioanalyzer RNA chip an RNA:RNA ratio of >10X for 18S peak in cytoplasm over nucleus. These two ratios for the same samples imply good nuclear and cytoplasmic partitioning.

**Figure 2:**
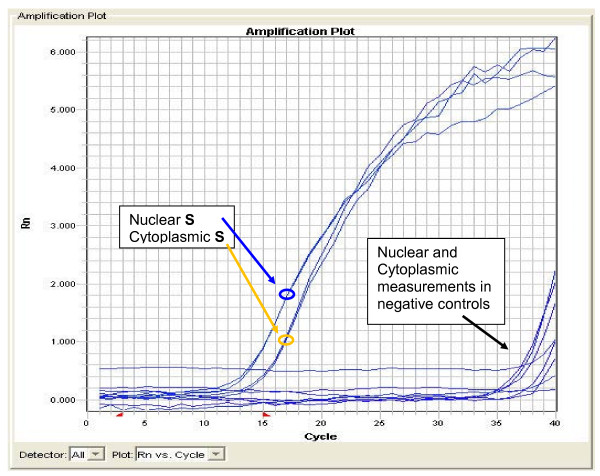
**A PCR assay for the 16-nt RNA molecule S = UUCGCGCUUUCCCCUG**. Two nuclear extracts and two cytoplasmic extracts from hNP1 human neural progenitor cells were assayed for the 16-nt sequence **S**. The two nuclear Rn values at 17 PCR cycles were about 1.7 versus the two cytoplasmic Rn values about 1.0. (Rn is the ratio of fluorescence emission intensity of the reporter dye divided by the fluorescence emission intensity of a passive reference dye.) Two types of negative controls were not detected in both nuclear and cytoplasmic fractions (see text).

We used a Qiagen (Alameda CA) miScript PCR system with custom primer to determine the presence of **S **in the samples. For negative control, we used a miScript primer for a 16-nt subsequence *Arabidopsis thaliana *miRNA-159a (mature previously shown by us to be absent in nuclear and cytoplasmic extracts from the same cell type, data not shown) and a 16-nt subsequence of human miRNA-128 (mature previously shown by us to be present in both nuclear and cytoplasmic fractions, data not shown). Input RNA templates were 0.76 pg/uL for nuclear extract and 1.01 pg/uL for cytoplasmic extract. All 12 samples were run simultaneously using an Applied Biosystems (Foster City CA) 7900HT Fast Real-Time PCR System. As shown in Figure 2, we readily and consistently detected **S **in both nuclear and cytoplasmic fractions from hNP1 cells. All negative controls were not detected or very weakly detected (cycle count >35).

### Methods of RNA targeting analysis

**Figure 3:**
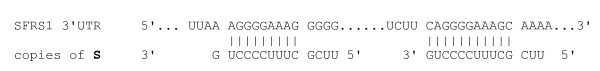
**Two putative targeting relationships between S and SFRS1**. Shown are alignments of two putative targeting relationships between the discovered sequence S and the 3'UTR of SFRS1.

Using TargetScan 5.1 [35] we found the *in silico *predicted targets for all ten of the seven-nt subsequences of **S**. Repeatedly appearing was SFRS1. This was because 11 consecutive nts from **S **were the exact reverse complement of a 3'UTR sequence of SFRS1; a second target was generated from 9 nts. How **S **might bind to the 3'UTR is shown in Figure 3; high C-G bond content suggests strong affinity.

We note that with one exception (miR-4315 with seed CGCUUUC) no seven-nt subsequence of **S **is also the seed of any human miRNA (nts 2-8 of all 1,100 miRBase v15 mature miRNAs [36]); thus targeting by **S **would likely not be redundant to conventional miRNA actions. Also to be noted is the fact that overall alignments are well within the parameters of potential alignments as reported by Thomas et al. [37].

SFRS1 protein is employed in protein-protein interactions and other processes and in particular recruits the U1 snRNP to the 5' splice site [38,39]. The upshot is the suggestion that a byproduct of RNU1 transcription is auto-regulation of spliceosome assembly and function.

Importantly, Ohrt et al. [40] have reported evidence of a nuclear RISC imported from the cytoplasm and consisting of Ago2 and a mature miRNA, thus some 20X smaller than the conventional cytoplasmic RISC. It therefore is possible that the small sequence **S **is also mounted in a nuclear RISC that includes Ago2.

### Methods of RNA folding analysis

**Figure 4:**
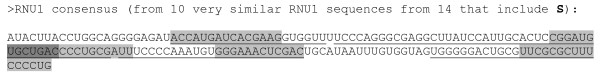
**Layout of predicted hairpins and observed cores in an RNU1 consensus sequence**. Underlined sequences correspond to predicted 2D hairpins, as declared by mfold and approximately as predicted and described by O'Gormann et al. [2]. Nucleotides highlighted in gray correspond to our declared "cores" of Illumina sequences (abundant shared subsequences in Illumina reads). In Sample 1, all cores but **S **had 45 to 154 instances, but **S **itself had 4736 instances. In the other two samples the counts were: 10-59 vs. 1317; 60-247 vs. 2454. In short, **S **was much more popular than any other sequenced cores apparently derived from RNU1.

**Figure 5:**
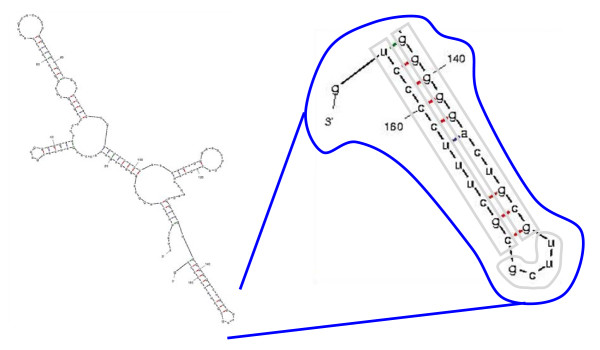
**One of the 2D structures of RNU1 predicted by mfold **[28]. Although other regions of RNU1 have alternative conformations, the hairpin designated herein as **H **and shown circled in blue is consistently present.

RNA hairpin structures are key features of RNA functions and processing generally, and miRNA processing in particular. The mfold engine uses dynamic programming and large tables of empirically derived binding affinities for small dsRNAs [28]. mfold predicts four hairpins formed from the RNU1 consensus (shown in Figure 4). mfold also predicts five 2D conformations for the full RNU1 sequence, four of which include the hairpin **H **with **S **as loop and 3' side; one full conformation is shown in Figure 5.

Regarding stability of the various hairpins, the stability ratio defined by -Gibbs free energy divided by number of nts (length) of a hairpin is -dG/L. For the fourth hairpin **H **in isolation, the number is much stronger (.59) than that of any other RNU1 hairpin (average ~.35).

### Methods of bioinformatic control mechanism analysis

**Figure 6:**
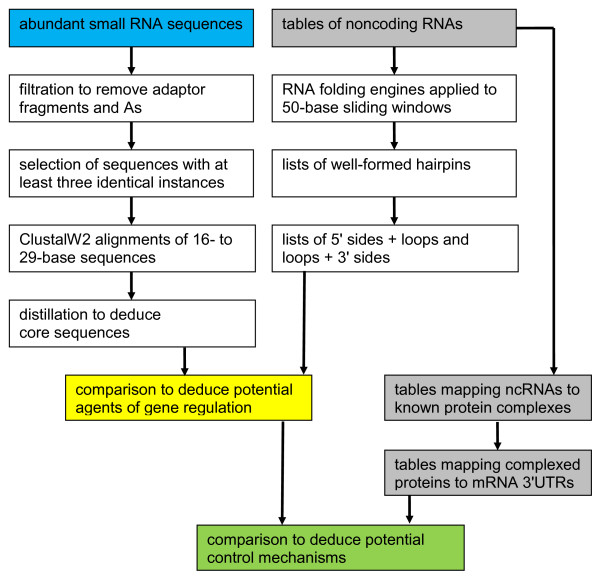
**Network concepts embedded in a flowchart**. The goal is efficient integration of selected types of information from sequencing, RNA folding, and gene function data. Sequencing reveals abundant small RNA sequences (blue box). Inputs for bioinformatic assays are tables of ncRNAs, proteins with which ncRNAs are complexed to yield gene processing moieties, and 3'UTR sequences of the mRNAs for the same proteins (gray boxes). Then biochemical assays and bioinformatic assays are compared to suggest agents of regulation (yellow box) and control mechanisms (green box).

Again, integration of biochemical and bioinformatic data to yield information about mechanisms of control of gene expression is the goal of this paper. To that end, the above example provides a paradigm for seeking causal relationships among biochemical concepts and bioinformatic concepts. Figure 6 will be described below to substantiate the paradigm.

Deep sequencing applied to cDNA libraries constructed from small RNA molecules provided us with millions of reads of observed sequences. From tables of Illumina 35-base reads, we trimmed from 3' regions the 5' ends of adaptors (at least six bases) and zero to four As, and then retained only the results that appeared at least three times. In our procedure, this yielded thousands of distinct sequences of 16 to 29 bases, each with an instance count ranging from three to more than 100,000. The sequences were aligned with ClustalW2, and then commonly occurring subsequences of at least 16 bases were deduced, each with a total instance count; we called each such subsequence a "core." This procedure includes multiple tuned parameters and an inevitable degree of arbitrariness. Conclusions reached with an algorithm containing tunable parameters must always be tested for invariance with respect to reasonable retuning.

In a separate line of investigation, tables of ncRNAs can be processed by submitting sliding windows of 50-nt subsequences to RNA folding engines such as mfold. Sought are simple RNA hairpins incorporating at least 25 nts and meeting certain stability criteria. For example, the above ratio of -Gibbs free energy (kcal/mol) to length (number of nts) in a hairpin should be at least ~0.30. A more sophisticated approach might include the minimal folding free energy index (MFEI) defined by Zhang et al. [41] as:

$$\text{MFEI}=\frac{-\text{Gibbs free energy }\left(\text{kcal}/\text{mol}\right)}{\text{number of G and C nts in hairpin}}$$

MFEI might be a criterion for finding RNA hairpins that are processed into mature miRNAs or other RNAs similar to mature miRNAs. A survey by Zhang et al. of 513 pre-miRNAs found average MFEI was 0.97, significantly higher than sampled mRNAs (0.65), tRNAs (0.64), or rRNAs (0.59). The distinguished hairpin in Figure 5 has MFEI = 0.85; all other predicted hairpins from RNU1 have MFEI ≤ 0.71.

Thus sequencing and hairpin searches can lead to comparisons of cores with sides of predicted hairpins to deduce potential agents of gene regulation, possibly processed in an miRNA-like manner.

In a third line of investigation, ncRNAs can be considered for membership in well annotated ribonucleoprotein particles of many types with wide-ranging functions [42]. The proteins in the same particles can be examined for subsequences in the 3'UTRs of their mRNAs that align with the putative miRNA-like agents formed from processed cores.

The complete flowchart of the proposed triple colligation is shown in Figure 6.

## Authors' contributions

All three contributed to the preparation of the manuscript and to various applications of bioinformatic analysis. CDJ conceived an initial version of the program for finding read cores and aligned some cores with known ncRNAs including RNU1; DOP contributed design of sequencing procedures and verification methods; XG contributed invention of data processing methods and programs. All authors read and approved the final manuscript.

## References

[B1] Staley JP Guthrie C Mechanical devices of the spliceosome: motors, clocks, springs, and things Cell 1998 92 315 326 10.1016/S0092-8674(00)80925-3 9476892

[B2] O'Gorman W Thomas B Kwek KY Furger A Akoulitchev A Analysis of U1 small nuclear RNA interaction with cyclin H The Journal of Biological Chemistry 2005 280 36920 36925 16115885 10.1074/jbc.M505791200

[B3] Kawaji H Nakamura M Takahashi Y Sandelin A Katayama S Fukuda S Daub CO Kai C Kawai J Yasuda J Carninci P Hayashizaki Y Hidden layers of human small RNAs BMC Genomics 2008 9 157 10.1186/1471-2164-9-157 18402656 PMC2359750

[B4] Liao JY Ma LM Guo YH Zhang YC Zhou H Shao P Chen YQ Qu LH Deep sequencing of human nuclear and cytoplasmic small RNAs reveals an unexpectedly complex subcellular distribution of miRNAs and tRNA 3' trailers PloS One 5 e10563 10.1371/journal.pone.0010563 20498841 PMC2871053

[B5] Shi W Hendrix D Levine M Haley B A distinct class of small RNAs arises from pre-miRNA-proximal regions in a simple chordate Nature Structural & Molecular Biology 2009 16 183 189 10.1038/nsmb.1536 PMC2746024 19151725

[B6] Langenberger D Bermudez-Santana C Hertel J Hoffmann S Khaitovich P Stadler PF Evidence for human microRNA-offset RNAs in small RNA sequencing data Bioinformatics (Oxford, England) 2009 25 2298 2301 10.1093/bioinformatics/btp419 19584066

[B7] Taft RJ Glazov EA Cloonan N Simons C Stephen S Faulkner GJ Lassmann T Forrest AR Grimmond SM Schroder K Irvine K Arakawa T Nakamura M Kubosaki A Hayashida K Kawazu C Murata M Nishiyori H Fukuda S Kawai J Daub CO Hume DA Suzuki H Orlando V Carninci P Hayashizaki Y Mattick JS Tiny RNAs associated with transcription start sites in animals Nature Genetics 2009 41 572 578 10.1038/ng.312 19377478

[B8] Taft RJ Glazov EA Lassmann T Hayashizaki Y Carninci P Mattick JS Small RNAs derived from snoRNAs RNA (New York, NY) 2009 15 1233 1240 10.1261/rna.1528909 PMC2704076 19474147

[B9] Taft RJ Simons C Nahkuri S Oey H Korbie DJ Mercer TR Holst J Ritchie W Wong JJ Rasko JE Rokhsar DS Degnan BM Mattick JS Nuclear-localized tiny RNAs are associated with transcription initiation and splice sites in metazoans Nature Structural & Molecular Biology 17 1030 1034 10.1038/nsmb.1841 20622877

[B10] Ender C Krek A Friedländer MR Beitzinger M Weinmann L Chen W Pfeffer S Rajewsky N Meister G A human snoRNA with microRNA-like functions Molecular Cell 2008 32 519 528 10.1016/j.molcel.2008.10.017 19026782

[B11] Saraiya AA Wang CC snoRNA, a novel precursor of microRNA in Giardia lamblia PLoS Pathogens 2008 4 e1000224 10.1371/journal.ppat.1000224 19043559 PMC2583053

[B12] Li Z Kim SW Lin Y Moore PS Chang Y John B Characterization of viral and human RNAs smaller than canonical MicroRNAs Journal of Virology 2009 83 12751 12758 10.1128/JVI.01325-09 19812168 PMC2786840

[B13] Langenberger D Bermudez-Santana CI Stadler PF Hoffmann S Identification and classification of small RNAs in transcriptome sequence data Pacific Symposium on Biocomputing 80 87 19908360 10.1142/9789814295291_0010

[B14] Haussecker D Huang Y Lau A Parameswaran P Fire AZ Kay MA Human tRNA-derived small RNAs in the global regulation of RNA silencing RNA (New York, NY) 16 673 695 10.1261/rna.2000810 PMC2844617 20181738

[B15] van Zon A Mossink MH Schoester M Scheffer GL Scheper RJ Sonneveld P Wiemer EA Multiple human vault RNAs. Expression and association with the vault complex The Journal of Biological Chemistry 2001 276 37715 37721 10.1074/jbc.M106055200 11479319

[B16] Stadler PF Chen JJ Hackermüller J Hoffmann S Horn F Khaitovich P Kretzschmar AK Mosig A Prohaska SJ Qi X Schutt K Ullmann K Evolution of vault RNAs Molecular Biology and Evolution 2009 26 1975 1991 10.1093/molbev/msp112 19491402

[B17] Persson H Kvist A Vallon-Christersson J Medstrand P Borg A Rovira C The non-coding RNA of the multidrug resistance-linked vault particle encodes multiple regulatory small RNAs Nature Cell Biology 2009 11 1268 1271 10.1038/ncb1972 19749744

[B18] Guengerich FP Cytochromes P450, drugs, and diseases Molecular Interventions 2003 3 194 204 10.1124/mi.3.4.194 14993447

[B19] Smalheiser NR Lugli G Thimmapuram J Cook EH Larson J Endogenous siRNAs and noncoding RNA-derived small RNAs are expressed in adult mouse hippocampus and are up-regulated in olfactory discrimination training RNA (New York, NY) 2010 in press 10.1261/rna.2123811 PMC3004058 21045079

[B20] Sossin WS Isoform specificity of protein kinase Cs in synaptic plasticity Learning & Memory (Cold Spring Harbor, NY) 2007 14 236 246 10.1101/lm.469707 17404386

[B21] Mermoud JE Cohen PT Lamond AI Regulation of mammalian spliceosome assembly by a protein phosphorylation mechanism The EMBO Journal 1994 13 5679 5688 7988565 10.1002/j.1460-2075.1994.tb06906.x PMC395533

[B22] Misteli T RNA splicing: What has phosphorylation got to do with it? Current Biology 1999 9 R198 200 10.1016/S0960-9822(99)80128-6 10209090

[B23] Kohtz JD Jamison SF Will CL Zuo P Luhrmann R Garcia-Blanco MA Manley JL Protein-protein interactions and 5'-splice-site recognition in mammalian mRNA precursors Nature 1994 368 119 124 10.1038/368119a0 8139654

[B24] Hoffmann S Otto C Kurtz S Sharma CM Khaitovich P Vogel J Stadler PF Hackermuller J Fast mapping of short sequences with mismatches, insertions and deletions using index structures PLoS Computational Biology 2009 5 e1000502 10.1371/journal.pcbi.1000502 19750212 PMC2730575

[B25] Friedländer MR Chen W Adamidi C Maaskola J Einspanier R Knespel S Rajewsky N Discovering microRNAs from deep sequencing data using miRDeep Nature Biotechnology 2008 26 407 415 18392026 10.1038/nbt1394

[B26] Zhu E Zhao F Xu G Hou H Zhou L Li X Sun Z Wu J mirTools: microRNA profiling and discovery based on high-throughput sequencing Nucleic Acids Research 38 Suppl W392 397 20478827 10.1093/nar/gkq393 PMC2896132

[B27] Gunaratne PH Creighton CJ Watson M Tennakoon JB Large-scale integration of microRNA and gene expression data for identification of enriched microRNA-mRNA associations in biological systems Methods in Molecular Biology (Clifton, NJ) 667 297 315 full_text 10.1007/978-1-60761-811-9_20 20827542

[B28] Zuker M Mfold web server for nucleic acid folding and hybridization prediction Nucleic Acids Research 2003 31 3406 3415 10.1093/nar/gkg595 12824337 PMC169194

[B29] Nakata K Lipska BK Hyde TM Ye T Newburn EN Morita Y Vakkalanka R Barenboim M Sei Y Weinberger DR Kleinman JE DISC1 splice variants are upregulated in schizophrenia and associated with risk polymorphisms Proceedings of the National Academy of Sciences of the United States of America 2009 106 15873 15878 10.1073/pnas.0903413106 19805229 PMC2736903

[B30] Atz ME Rollins B Vawter MP NCAM1 association study of bipolar disorder and schizophrenia: polymorphisms and alternatively spliced isoforms lead to similarities and differences Psychiatric Genetics 2007 17 55 67 10.1097/YPG.0b013e328012d850 17413444 PMC2077086

[B31] Larkin MA Blackshields G Brown NP Chenna R McGettigan PA McWilliam H Valentin F Wallace IM Wilm A Lopez R Thompson JD Gibson TJ Higgins DG Clustal W and Clustal × version 2.0 Bioinformatics 2007 23 2947 8 10.1093/bioinformatics/btm404 17846036

[B32] Creighton CJ Reid JG Gunaratne PH Expression profiling of microRNAs by deep sequencing Briefings in Bioinformatics 2009 10 490 497 10.1093/bib/bbp019 19332473 PMC2733187

[B33] Dhara SK Hasneen K Machacek DW Boyd NL Rao RR Stice SL Human neural progenitor cells derived from embryonic stem cells in feeder-free cultures Differentiation; Research in Biological Diversity 2008 76 454 464 18177420 10.1111/j.1432-0436.2007.00256.x

[B34] Dhara SK Gerwe BA Majumder A Dodla MC Boyd NL Machacek DW Hasneen K Stice SL Genetic manipulation of neural progenitors derived from human embryonic stem cells Tissue Engineering 2009 15 3621 3634 10.1089/ten.tea.2009.0155 19795983 PMC2792066

[B35] Friedman RC Farh KK Burge CB Bartel DP Most mammalian mRNAs are conserved targets of microRNAs Genome Research 2009 19 92 105 10.1101/gr.082701.108 18955434 PMC2612969

[B36] Griffiths-Jones S Saini HK van Dongen S Enright AJ miRBase: tools for microRNA genomics Nucleic Acids Research 2008 36 Database D154 158 17991681 10.1093/nar/gkm952 PMC2238936

[B37] Thomas M Lieberman J Lal A Desperately seeking microRNA targets Nature Structural & Molecular Biology 17 1169 1174 10.1038/nsmb.1921 20924405

[B38] Jamison SF Pasman Z Wang J Will C Luhrmann R Manley JL Garcia-Blanco MA U1 snRNP-ASF/SF2 interaction and 5' splice site recognition: characterization of required elements Nucleic Acids Research 1995 23 3260 3267 10.1093/nar/23.16.3260 7667103 PMC307186

[B39] Cao W Garcia-Blanco MA A serine/arginine-rich domain in the human U1 70 k protein is necessary and sufficient for ASF/SF2 binding The Journal of Biological Chemistry 1998 273 20629 20635 10.1074/jbc.273.32.20629 9685421

[B40] Ohrt T Mutze J Staroske W Weinmann L Hock J Crell K Meister G Schwille P Fluorescence correlation spectroscopy and fluorescence cross-correlation spectroscopy reveal the cytoplasmic origination of loaded nuclear RISC in vivo in human cells Nucleic Acids Research 2008 36 6439 6449 10.1093/nar/gkn693 18842624 PMC2582625

[B41] Zhang BH Pan XP Cox SB Cobb GP Anderson TA Evidence that miRNAs are different from other RNAs Cell and Molecular Life Sciences 2006 63 246 254 10.1007/s00018-005-5467-7 PMC11136112 16395542

[B42] Dreyfuss G Philipson L Mattaj IW Ribonucleoprotein particles in cellular processes The Journal of Cell Biology 1988 106 1419 1425 10.1083/jcb.106.5.1419 2836428 PMC2115050

